# Graphene-Assisted Electrochemical Sensor for Detection of Pancreatic Cancer Markers

**DOI:** 10.3389/fchem.2021.733371

**Published:** 2021-08-19

**Authors:** Zhenglei Xu, Minsi Peng, Zhuliang Zhang, Haotian Zeng, Ruiyue Shi, Xiaoxin Ma, Lisheng Wang, Bihong Liao

**Affiliations:** ^1^ Department of Gastroenterology, The Second Clinical Medical College, Shenzhen People’s Hospital, Jinan University, Shenzhen, China; ^2^ Department of Cardiology, Shenzhen People’s Hospital, The Second Clinical Medical College of Jinan University, Shenzhen, China

**Keywords:** pancreatic cancer, microRNA-21, microRNA-196a, CEA, signal amplification, k-ras gene

## Abstract

Pancreatic cancer is a highly lethal gastrointestinal malignancy. Most patients are already in the middle to advanced stages of pancreatic cancer at the time of diagnosis and cannot be treated completely. As a single-atom planar two-dimensional crystal, graphene’s unusual electronic structure, specific electronic properties and excellent electron transport capacity make it uniquely advantageous in the field of electrochemical sensing. In this mini-review, we summarize the potential application of graphene in pancreatic cancer detection. K-Ras gene, CEA and MicroRNA are important in the early diagnosis of pancreatic cancer.

## Introduction

Pancreatic cancer is a malignant tumor of the gastrointestinal tract with a high mortality rate. Currently, the diagnosis and treatment of pancreatic cancer are difficult (Neoptolemos et al., 2018). Since the clinical symptoms of early stage pancreatic cancer are not obvious, most patients are already in the middle and late stage of pancreatic cancer when they are diagnosed, and the 1-year survival rate is less than 20%. The 5-years survival rate of pancreatic cancer patients is still less than 3% with the most effective treatment methods available. The 5-years survival rate for patients with advanced pancreatic cancer is almost zero (Rawla et al., 2019). Considering the high mortality rate of pancreatic cancer, the diagnosis of pancreatic cancer at an early stage will significantly reduce the mortality rate of pancreatic cancer (Singhi et al., 2019).

A suitable tumor marker should be highly specific, sensitive and easy to detect. However, numerous clinical studies have shown that it is very difficult to find an ideal tumor marker with 100% specificity and sensitivity and whose level correlates with tumor size, stage and prognosis (Lai et al., 2018). In the clinical diagnosis, some tumor markers with high specificity and complementary tumor detection are often selected to improve the detection rate of tumors (Huang et al., 2018b). Electrochemical sensor devices are an important tool for gene structure analysis and detection. It can be used for rapid identification and detection of specific gene sequences by taking advantage of the specific complementary pairing laws between molecules (Shan and Ma, 2017; Li et al., 2021; Yan et al., 2021).

Graphene is a new type of carbon nanomaterial discovered in 2004. With its ideal flat two-dimensional structure, unique electronic, thermal, optical and mechanical properties, it has excellent prospects for high-tech applications in electronics, mechanics, medicine and aerospace. After carbon nanotubes, it is an emerging carbon nanomaterial with great theoretical and application prospects after carbon nanotubes. As a single-atom planar two-dimensional crystal, graphene’s unusual electronic structure, specific electronic properties and excellent electron transport capacity make it uniquely advantageous in the field of high-sensitivity detection (Akbari jonous et al., 2019). Graphene, on the one hand, combines the redox properties of some bioelectrically active molecules and can detect the target molecule by its redox reaction on the electrode surface and the corresponding current signal (Coroş et al., 2019; Wang et al., 2021b). On the other hand, the bipolarity of graphene can be easily monitored in resistive sensors (Krishnan et al., 2019). Graphene is thus expected to allow highly sensitive monitoring, even of individual molecules adsorbed to or leaving the surface of graphene (Thangamuthu et al., 2019).

In this mini-review, we summarize the potential application of graphene in pancreatic cancer detection. K-Ras gene, CEA and MicroRNA are important in the early diagnosis of pancreatic cancer. Therefore, this mini-review was divided into three sections. Each section first described the value of the clinical application of the analyte. Then, we described how the electrochemical sensor can achieve sensitive detection with the assistance of graphene.

### Mutant K-Ras Gene Detection

A large number of studies have shown that the mutation rate of K-Ras gene in pancreatic cancer patients can reach more than 90%, and the product of K-Ras gene expression is K-Ras protein with GTPase activity (Jelski and Mroczko, 2019). The K-Ras gene expression product is K-Ras protein, which has GTPase activity. It is activated when it binds to GTP and inactivated when it binds to GDP (Satoh, 2021). K-Ras proteins are mainly located on the cell membrane. PKC phosphorylates K-Ras protein, which weakens its binding to the cell membrane and leads to a change in location (Meng et al., 2020). K-Ras proteins also act as molecular switches and play important roles in many signaling pathways (Wang et al., 2017). The 12 codon of wild-type K-Ras gene is GGT, which can be mutated to GAT, GTT and GCT. The common mutation type is mainly GAT, and the mutation rate can reach more than 52% (Wang et al., 2019). K-Ras gene mutations are a typical genetic point mutation, and mutant K-Ras genes can be detected in the early stages of pancreatic cancer. Therefore, the mutant K-Ras gene can be a landmark gene for early pancreatic cancer diagnosis (Van Sciver et al., 2018). Detection of mutations in the K-Ras gene enables early pancreatic cancer surveillance and screening. DNA electrochemical sensing electrodes are an important tool for gene structure analysis and detection (Park et al., 2017). It enables the rapid identification and detection of specific gene sequences by exploiting the specific complementary pairing pattern between DNA molecules.

Shu et al. (2020) assembled an electrochemical sensor for efficient and ultrasensitive detection of K-Ras gene fragments using exonuclease III-assisted targeting cycling and π-π stacking between graphene and nucleotide bases. Since the sensor utilized fully complementary target DNA, exonuclease III could trigger the target DNA loop, forming a short single-stranded DNA probe. At the same time, they synthesized a novel ferrocene as a probe for providing an electrochemical signal. This sensor showed an excellent K-Ras detection capability and could reach a detection limit of 20.4 fM.

Graphene quantum dots (GQDs) were used for electrochemiluminescence (ECL) detection of K-Ras genes (Zhang et al., 2020). To improve the quantum yield of GQDs, nitrogen (N-GQDs) was doped in GQDs. This can make the ECL efficiency of N-GQDs greatly improved. Deoxyribonucleic acid (DNA) was used as a ligating medium to adjust the distance between GQDs and AuNPs. DNA double-stranded hybridization was reached after 1 h incubation with the target K-Ras DNA. The assay results showed that this GQDs-based composite could be used for quantitative determination of K-Ras.

### MicroRNA Detection

Recent studies have found that aberrant expression of microRNA, a highly conserved class of endogenous non-coding RNA molecules of 18–25 nucleotides in length, is also associated with pancreatic cancer (Kanno et al., 2019; Mohammadi et al., 2019). It is widely present in blood and can regulate gene expression at both transcriptional and post-transcriptional levels. When microRNAs with tumor suppressor effects are formed during the process of gene mutation, deletion, promoter methylation and other changes, their expression is down-regulated or loss of function cannot down-regulate oncogenes normally (Moutinho-Ribeiro et al., 2019; Pang et al., 2019). For example, microRNA-15a, microRNA-16 and Let-7a are down-regulated and their target oncogenes (e.g., BCL-2, RAS) were abnormally expressed, leading to malignant tumorigenesis. When microRNAs with oncogenic effects are overexpressed, they downregulate tumor suppressors or involve other unrelated genes in cell differentiation (Si et al., 2020). Overexpression of oncogenic microRNA-17, microRNA-21 and microRNA-210 downregulates tumor suppressors (e.g., TGFBR2), leading to the appearance of malignant tumors (Bartsch et al., 2018). The abnormal expression of microRNA is closely related to the occurrence, development and metastasis of pancreatic cancer, especially miR-21 is overexpressed in the blood of pancreatic cancer patients (Shen et al., 2018). Moreover, miR-21 can be used to distinguish cancerous pancreas from normal pancreas and also as an indicator of pancreatic cancer prognosis.

Kilic et al. (2015) performed the first electrochemical detection of miR-21 in cell lysates using a graphene-modified disposable pencil-graphite electrode (GME). The surface characteristics of GME were analyzed by electrochemical impedance spectroscopy (EIS) and scanning electron microscopy (SEM). The detection limit of the sensor was reduced by 2.77-fold to 2.09 µg/ml (3.12 pM) by the modification of graphene. In addition, the results of this sensor were used to analyze mir-21 in cell lysates of miR-21 positive breast cancer cell line (MCF-7) and miR-21 negative hepatocellular carcinoma cell line (HUH-7).

Shin Low et al. (2020) proposed a smartphone-based biosensing system for the detection of miR-21 in saliva. reduced graphene oxide/gold (rGO/Au) composite modified screen-printed electrodes are the main body of the sensor. miR-21 and ssDNA probes hybridize and the peak current decreases. Under optimal conditions, this sensor can sub ah between 1 pM and 0.1 mM for miR-21 detection. In addition, Zhang et al. (2014) reported a selective and sensitive biosensing prototype based on graphene nanocomposite with functionalized AuNPs for the sensing of miR-21.

### CEA Detection

Carcino-embryonic antigen (CEA), which is widely present in digestive system cancers of endodermal origin, is also present in the digestive tract of normal embryos (Jing et al., 2020). CEA is one of the important markers of pancreatic cancer (Yang et al., 2019). The sensitivity of CEA for the diagnosis of pancreatic cancer is 55–78% and the specificity is 25–75% when the reference value is 2.5 ng/ml (Wang et al., 2021a). When the reference value is 5 ng/ml the sensitivity is 35–53% and the specificity is 32–80%. Currently, the main protein tumor marker for clinical detection of pancreatic cancer is CA19-9; however, CA19-9 is a sialic acid Lewis blood group antigen that may remain undetectable in Lewis-negative individuals with advanced pancreatic cancer (Rizwan et al., 2018). Compared with CA19-9, CEA does not have this problem, so CEA was selected as a serum marker for pancreatic cancer. However, as a tumor marker, the expression level of CEA increases in both upper gastrointestinal tumors and pancreatic cancer (Yue et al., 2021), and the level of CEA in some tumors is even higher than that in pancreatic cancer (Hasanzadeh et al., 2017; Karimi-Maleh et al., 2021a; Karimi-Maleh et al., 2021b; Karimi-Maleh et al., 2021c). The sensitivity and specificity of CEA alone for pancreatic cancer screening are insufficient to meet the clinical need for early pancreatic cancer screening (Wang et al., 2018). Therefore, CEA needs to be combined with pancreatic cancer-specific tumor markers (e.g., miR-21) for the diagnosis of early pancreatic cancer (Li et al., 2017).

Han et al. (2011) prepared a CEA electrochemical based on graphene and several other nanomaterials. They first modified chitosan, ferrocene, and nano-TiO_2_ (CS-Fc-TiO_2_) composite membranes onto GCE. Then, AuNPs and graphene nanohybrids were self-assembled onto the CS-Fc-TiO_2_ composite membrane modified electrode. This combination utilizes the strong interaction between AuNPs and carcinoembryonic antibody (anti-CEA) to further immobilize anti-CEA on the modified electrode surface, resulting in a CEA immunosensor. Due to the high surface free energy, strong adsorption affinity, good adaptability and conductivity of AuNPs, they provide more binding sites and a more suitable microenvironment for the immobilization of biomolecules. The synergistic effect of graphene and AuNPs leads to high conductivity and sensitivity, with linear detection range of 0.01–80 ng/ml for CEA and detection limit of 3.4 pg/ml (S/N = 3). The sensor showed excellent selectivity for CEA by detecting some possible interferents. The relative deviations of the results for six plasma samples were less than 10% compared with those of the commonly used clinical ELISA method. The good agreement between the two methods was obtained, and the simplicity and rapidity of the electrochemical assay showed great potential for its clinical application and for the detection of low levels of protein. Liu et al. (2017) constructed an electrochemical immunosensor for the detection of CEA with a three-dimensional structure of reduced graphene oxide-gold nano (3D RGO-Au) as the substrate material and Cu_2_SnZnS_4_ (CZTS) as the antibody marker material, with a wide linear range of 0.5 pg/ml to 20 ng/ml and a detection limit of 0.16 pg/ml.

Zhou et al. (2015) reported a novel nucleic acid aptamer/graphene oxide (Apt/GO)-based biosensor for CEA determination by capillary electrophoresis-chemiluminescence (CE-CL). After the CEA aptamer conjugated with horseradish peroxidase (HRP) is mixed with GO, the CL is quenched because the HRP-Apt stacked on GO leads to the chemiluminescence resonance energy transfer. In the presence of CEA, HRP-Apt and CEA form an HRP-Apt-CEA complex, resulting in the separation of the complex from GO. The CL catalyzed by the HRP-Apt-CEA complex can then be detected in the absence of any CRET. the CEA content is calculated from the CL intensity, which is linearly related to the CEA concentration from 0.0654 to 6.54 ng/ml, with a detection limit of 4.8 pg/ml.

Huang et al. (2018a) developed an electrochemical nucleic acid aptamer biosensor based on lead ion (Pb^2+^)-dependent DNA enzyme-assisted signal amplification and graphene quantum dot ionic liquid-ion (GQDs-IL-NF) composite membrane for highly sensitive determination of CEA. The CEA-containing nucleic acid aptamer and the hairpin DNA of the DNAzyme chain recognize the target and form a CEA-nucleic acid aptamer complex in the presence of CEA. In the presence of Pb^2+^, a DNAzyme-assisted signal amplification reaction was performed to produce large amounts of ssDNA. Adsorption of ssDNA onto GQDs-IL-NF-modified GCEs by π-π stacking. As a result, methylene blue-labeled substrate DNA (MB-substrate) is immobilized on the electrode and generates an electrochemical signal. Under optimal conditions, the biosensor shows a linear range of 0.5 fg/ml to 0.5 ng/ml with a detection limit of 0.34 fg/ml.

## Conclusion

Graphene, a novel material with a monolayer, two-dimensional carbon nanostructure, has a great specific surface area and excellent electronic, chemical, and mechanical properties. It also has excellent biocompatibility, which makes it extremely useful in all bioelectrical analyses. In the detection of pancreatic cancer, graphene-based biosensors can show excellent sensitivity and selectivity for both proteins (cancer markers) and DNA, making it an ideal material for the construction of efficient, fast, and sensitive detection biosensors. However, there are still some factors affecting the application of graphene in biosensors that need further detailed study. These issues include 1) the effect of the oxygen-containing functional group fraction in graphene on its electrochemical properties; 2) how to prepare graphene with high electrical conductivity and good solution dispersion; 3) the effect of heteroatom doping on the electrochemical properties and stability of graphene; 4) the connection mode and interaction between biomolecules and graphene in sensors; 5) the biocompatibility of graphene in different biosensing applications issues. These studies related to graphene-based materials will open new directions in the field of biosensor research.
